# Keep Cool

**Published:** 1913-06

**Authors:** 


### BUFFALO MEDICAL JOURNAL

**A Monthly Review of Medicine and Surgery**

EDITOR AND PUBLISHER DR. A. L. BENEDICT, 228 Summer St., omer of Elmwood Ave., Buffalo. (Address for all communications. Please make cpersonal and telephone calls before 1 P. M.)

THE BUFFALO MEDICAL JOURNAL is the third, in age, in the western continent. It is independent but supports professional organization. As a scientific medium it is unrestricted in territory. As a news medium, it is limited mainly to the western four districts of the Medical Society of the State of New York. The co-operation of physicians is asked in securing personal, obituary and other news items. Secretaries of Medical Societies in this region are requested to send brief reports of meetings. Full transactions will be published at cost of composition.

Subscription $2.00 a year in advance; $3.00 for two years in advance. Changes and errors in address or failure or duplication of delivery, should be reported immediately. Back numbers will be supplied if possible but requests for copies should be made promptly.

YEARLY VOLUME 68. JUNE, 1913 No. 11

*The cat, not highly esteemed for intelligence or altruism, digs a hole and covers it up. The dog, more intelligent and more influenced by man, has reduced his hereditary instinct to three inefficient kicks at the dirt. Man, till a hundred years ago, left it for his neighbor to step in; with greater enlightenment, he conveniently dropped it into his neighbor's water supply. Recently, very reluctantly and only in response to scientific demonstration regarding typhoid and cholera, he is beginning to manifest the instinctive decency of the cat.*

### Keep Cool.

The heat regulating function of the human body, in a natural state, is said to be competent to keep the individual fairly comfortable and free from danger from either hyperthermia or chilling, without clothing, for a range of 10 degrees centigrade either way from the normal, *i. e.,* from about 81 to 117 Fahrenheit. For this region the ordinary summer variation is from about 60 to 95.

With the customary clothing, the average person suffers from heat during much of the summer. His efficiency is lessened, he is tempted to the too free use of beverages more or less harmful in themselves, beside the possible danger of heat stroke, it is altogether likely that the continued discomfort and the disturbance of circulatory equilibrium and that normally attending the passage of water into and out of the body, may result in various visceral lesions. With due respect for the pneumococcus, the somewhat putative rheumatococcus and other germs, the almost inevitable diurnal lowering of temperature acting on a relaxed and moistened skin, is liable to result in various inflammatory disorders including one phase of the many sided symptom-complex still commonly called rheumatism.

The moral is, to dress as coolly as possible for the day and to carry extra wraps for the evening. Personally, we disagree entirely with those who advocate wearing flannels till the first day of May. Indeed, we think woolen underwear should be limited to elderly, sedentary, dry-skinned persons, and that cotton or other more hygroscopic garments should be worn, even in winter, by ordinary persons. It is a good rule to dress according to the thermometer and not the calendar.

As to the complicated problems of women's dress, we have nothing to say further than to express the hope that fashions may be modified so as to allow a more even distribution of heat-retaining devices. Still, there is something to be said in favor of the method of carefully preserving the body heat over the viscera and leaving the extremities in a state of nakedness or what approaches it.

As to men's wear, we offer the following suggestions: Wear low shoes and a straw hat or some other light head covering and keep it off whenever possible. Have the drawers loose, practically washable trouser-linings, coming far enough below the knees to prevent contact with the trousers. Suspend with a tape a handkerchief over the chest in lieu of an undershirt. Wear an outing shirt with a soft collar if you have the moral courage. Have the coat and trousers as light as possible and wear no vest (we beg pardon, waistcoat). Keep your coat off and your shirt-sleeves rolled up as much as convention allows. Even a fat man can wear a belt and dipense with suspenders, if his tailor knows enough to cut the trousers so as to engage at the illiac crest. Most of the energy wasted in keeping the trousers up is due to their being cut in the empire fashion. On hot days don't loll in a hammock with a fan and a pitcher of ice water, but get out and sweat. On excessively hot days, when there is no pleasure in this, stay indoors as much as you can in your birthday suit, visit the bath tub occasionally, and you will be surprised how much desk work you can accomplish.

